# Novel human neurodevelopmental and neurodegenerative disease associated with IRF2BPL gene variants—mechanisms and therapeutic avenues

**DOI:** 10.3389/fnins.2024.1426177

**Published:** 2024-06-06

**Authors:** Daniel Bauersachs, Louise Bomholtz, Sara del Rey Mateos, Ralf Kühn, Pawel Lisowski

**Affiliations:** ^1^Genome Engineering & Disease Models, Max Delbrück Center for Molecular Medicine in the Helmholtz Association (MDC), Berlin, Germany; ^2^Quantitative Stem Cell Biology, Berlin Institute for Medical Systems Biology (BIMSB) Max-Delbrück-Center for Molecular Medicine (MDC), Berlin, Germany; ^3^Department of Psychiatry, Neuropsychiatry Research Division, Translation and Neurotechnology Research Group, Charité—Universitätsmedizin Berlin, Berlin, Germany

**Keywords:** NEDAMSS, IRF2BPL, neurodevelopmental disorder, NDDs, gene therapy, rare genetic disorder

## Abstract

Recently a broad range of phenotypic abnormalities related to the neurodevelopmental and neurodegenerative disorder NEDAMSS (Neurodevelopmental Disorder with Regression, Abnormal Movements, Loss of Speech, and Seizures) have been associated with rare single-nucleotide polymorphisms (SNPs) or insertion and deletion variants (Indel) in the intron-less gene IRF2BPL. Up to now, 34 patients have been identified through whole exome sequencing carrying different heterozygous pathogenic variants spanning the intron-less gene from the first polyglutamine tract at the N-terminus to the C3HC4 RING domain of the C-terminus of the protein. As a result, the phenotypic spectrum of the patients is highly heterogeneous and ranges from abnormal neurocognitive development to severe neurodegenerative courses with developmental and seizure-related encephalopathies. While the treatment of IRF2BPL-related disorders has focused on alleviating the patient’s symptoms by symptomatic multidisciplinary management, there has been no prospect of entirely relieving the symptoms of the individual patients. Yet, the recent advancement of CRISPR-Cas9-derived gene editing tools, leading to the generation of base editors (BEs) and prime editors (PEs), provide an encouraging new therapeutic avenue for treating NEDAMSS and other neurodevelopmental and neurodegenerative diseases, which contain SNPs or smaller Indels in post-mitotic cell populations of the central nervous system, due to its ability to generate site-specific DNA sequence modifications without creating double-stranded breaks, and recruiting the non-homologous DNA end joining repair mechanism.

## 1 Introduction

In humans, mutations in IRF2BPL lead to a neurodevelopmental disorder called NEDAMSS (Neurodevelopmental Disorder with Regression, Abnormal Movements, Loss of Speech, and Seizures; OMIM-#618088). NEDAMSS is a new, ultra-rare, severe neurodegenerative disorder with neurological symptoms that deteriorate during infancy and early childhood. Patients with NEDAMSS initially develop normally before an eventual regression and loss of skills. Typical symptoms include loss of motor skills (e.g., crawling and walking), loss of speech, abnormal movements, and seizures. The disorder is caused by a variety of *de novo* genetic mutations of the Interferon Regulatory Factor 2 Binding-Like (IRF2BPL) gene (Marcogliese et al., 2018; Skorvanek et al., 2019; Tran Mau-Them et al., 2019).

IRF2BPL, located on chromosome 14q24.23, is an intron-less gene that encodes a 796 amino acids protein and a transcript of 4,166 nucleotides(ENSEMBL IRF2BPL [30.04.2024]) belonging to the IRF2BP family, where it acts as a transcriptional regulator localized almost exclusively to the nucleoplasm. (The Human Protein Atlas [30.04.2024]) Similar to its paralogues, IRF2BP1 and IRF2BP2, IRF2BPL contains two conserved domains: a coiled-coil DNA binding domain at its N-terminus and a C3HC4 RING finger domain at its C-terminus (Rampazzo et al., 2000), suggesting IRF2BPL transcriptional regulatory functions, either as a trans activator or repressor, depending on the target promoter and interacting partners (Shimono et al., 2000). Additionally, IRF2BPL contains polyalanine (Poly-A) and polyglutamine (Poly-Q) regions at its N-terminus, along with a Nuclear Localization Signal (NLS) towards the C-terminus. Notably, Poly-Q motifs are primarily found in transcription factors and serve as domains for regulating transcriptional processes by mediating interactions with other transcriptional regulators. IRF2BPL also contains multiple putative proline, glutamic acid, serine, and threonine-rich (PEST) sequences in the variable region between its highly conserved domains, suggesting the potential post-translational regulation of this protein (Marcogliese et al., 2018).

Currently, the precise biological function of IRF2BPL is unknown. Nevertheless, it has been associated with various physiological processes, whereas its involvement or role in both neuronal development and maintenance has recently gained awareness (Sabitha et al., 2021). The diagnosis of NEDAMSS patients typically begins with a physical examination. During this process, it is important to exclude conditions such as multi-organ involvement, behavioral/psychiatric problems, post-infectious autoimmune encephalitis, perinatal HIV infection, and infantile spasms to avoid misdiagnosis with the IRF2BPL variant syndrome. As trio-based whole exome sequencing (WES) or whole genome sequencing (WGS) is the gold standard for identifying the molecular etiologies of these disorders (Marcogliese et al., 2018; Shelkowitz et al., 2019; Tran Mau-Them et al., 2019; Prilop et al., 2020; Pisano et al., 2022), Sanger sequencing is often performed to confirm the underlying mutation in the IRF2BPL gene. This approach facilitated the diagnosis of the first two NEDAMSS patients in 2018, followed by the identification of an additional 32 patients to date. Brain Magnetic Resonance Imaging (MRI) is performed to check for abnormalities (Shelkowitz et al., 2019; Skorvanek et al., 2019; Tran Mau-Them et al., 2019; Ginevrino et al., 2020; Prilop et al., 2020; Pisano et al., 2022), where the majority of patients mainly display focal or diffuse cortical/subcortical atrophy, cerebellar atrophy, and thinning of the corpus callosum (Pisano et al., 2022). An electroencephalogram (EEG) is also regularly performed (Marcogliese et al., 2018; Tran Mau-Them et al., 2019; Ginevrino et al., 2020; Pisano et al., 2022) since many patients tend to suffer from seizures or have epileptic manifestations, due to spontaneous electrical activities in the brain, including irregular theta activity (Skorvanek et al., 2019).

However, comprehension of the molecular characteristics of IRF2BPL-related disorders presents several key challenges due to the absence of a significant association between the genetic variants with the severity of the phenotype and the age of onset. This limitation is further enhanced by the restricted availability of neural tissues for in-depth study, a lack of comprehensive information regarding the disease’s natural progression, the rarity of affected individuals, and the absence of specific *in vitro* and animal models for research purposes (Sabitha et al., 2021). Yet the accelerated advances in genome sequencing have emerged with a greater awareness of the human genome in disease prevention and treatment of individual patients, contrasting the current focus on alleviating the patient’s symptoms by symptomatic multidisciplinary management. This revolution is tightly linked to CRISPR-Cas9-derived technologies such as base- and prime editors, which have expanded the therapeutic potential of genomic engineering in neuronal and non-neuronal cell populations of the CNS, potentially leading to the correction of the pathogenic variants that cause NEDAMSS in patients in the future.

## 2 IRF2BPL function in neurodevelopmental and neurodegenerative disorders

**Figure 1:**
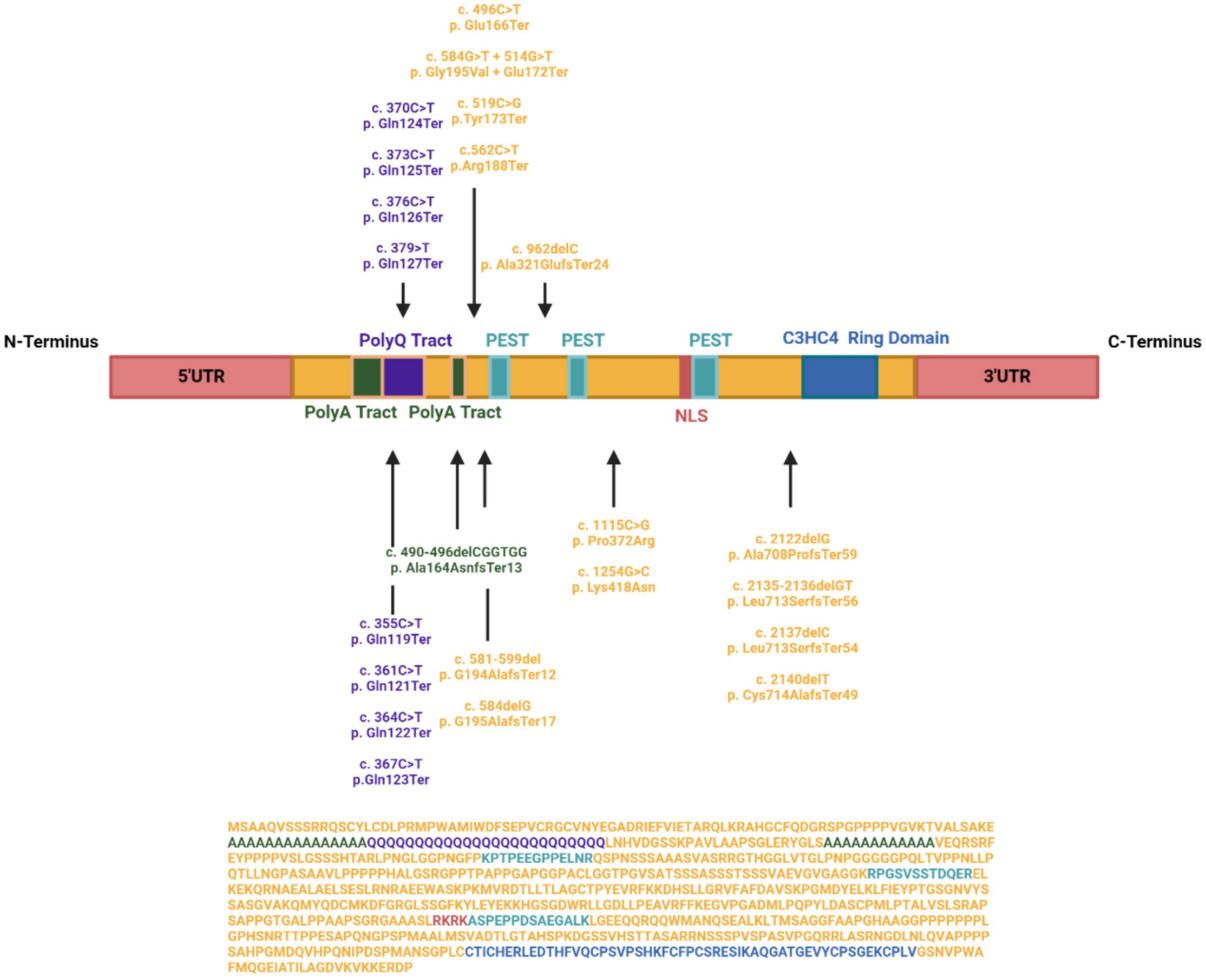
IRF2BPL Landscape. Distribution of known mutations across the length of IRF2BPL, including known functional domains. Mutations are named for the resultant nucleotide change, as well as the affected amino acid residue (first letter), position (number), and the resultant change to the residue due to the mutation (following letter).

Clinical reports have documented 34 patients with pathogenic variants, constituting 19 nonsense, 2 missense, and 12 frameshift variants across IRF2BPL with 1 patient having both a nonsense and missense mutation in the gene (Figure 1). In particular, nonsense variants display the most severe developmental disability (Tran Mau-Them et al., 2019). Yet no significant association and correlation have been established between the location of the pathogenic variations and the severity of the phenotype (Pisano et al., 2022), which may be due to the limited number of documented cases, aligning with the absence of therapeutic avenues for NEDAMSS or the role of the patient’s genetic background.

The establishment of an appropriate model system for NEDAMSS to shed additional light on the biological function of IRF2BPL is therefore crucial, leading to the generation of Drosophila, zebrafish, and *in vitro* human models.

## 3 Model organisms

Various heterogeneous *de novo* mutations have been observed in IRF2BPL, resulting in characteristic phenotypes depending on the induced variant in the NEDAMSS patients. Ubiquitous overexpression of the reference IRF2BPL or Pits in Drosophila at low temperatures (Act-Gal4 at 18°C, PitsTG4/+) caused lethality. The same was observed upon the null mutation of Pits, resulting in unsuccessful survival past the early larval stage or even embryonic lethality, suggesting the gene is very dosage sensitive and greatly regulated *in vivo*. However, overexpression of three nonsense variants (Gln127*, Glu172*, and Arg188*) generating a premature termination of translation did not display any toxicity, whereas the missense variants Lys418Arg and Pro372Arg displayed various effects upon overexpression, indicating the position and amino acid change impact the protein sequence toxicity upon overexpression. Additionally, the reduction of Pits in the fruit fly, mimicking the nonsense IRF2BPL variants in NEDAMSS patients resulted in a bang-sensitive phenotype and a progressive decline in climbing activity only seen in adulthood flies, which is particularly similar to the seizures-like paralysis, known as epilepsy and progressive motor dysfunction observed in adolescence patient with NEDAMSS. Reduction of Pits also affected the photoreceptors, leading to a decrease and age-dependent deficit of neuronal integrity, equivalent to the cerebral symptoms and atrophy observed in patients. This indicates the requirement of Pits expression in the neurons of the antennal mechanosensory and motor center to acquire accurate balance, auditory and motor coordination, supporting the essential function of IRF2BPL and Pits in the development and maintenance of the CNS (Marcogliese et al., 2018).

Recently, a covalent interaction was identified between Pits/irf2bpl/IRF2BPL and wg/wnt1/WNT1 in Drosophila, zebrafish, and humans with the reduction of Pits/irf2bpl/IRF2BPL, leading to an increase in wg/wnt1/WNT1 transcript and protein expression. Consequently, the partial loss of Pits in neurons of adult flies, mimicking the IRF2BPL nonsense allele variations, displayed a shortened lifespan, progressing climbing defects, and progressive degradation of the peripheral axons, normally associated with overexpression of wg in flies. Yet, reversion of the neurobehavioral phenotype was observed by the overexpression of Casein kinase 1 alpha (CK1α) (CSNK1A1 in humans) (Marcogliese et al., 2022), a regulator of signal transduction pathways, playing a significant role in the WNT signaling pathway through phosphorylation of β-catenin (Jiang et al., 2018), which IRF2BPL is known to targeting for proteasomal degradation (Higashimori et al., 2018). As a result, IRF2BPL is implied to interact and colocalize with CSNK1A1 in the cytoplasm, promoting the entering into the nucleus, where the complex acts as a strong transcriptional repressor of the Wnt/β-catenin signaling pathway (Marcogliese et al., 2022).

## 4 Human astrocytes/neurons

To draw a parallel between the phenotypic changes observed within Drosophila and Zebrafish, with the orthologous proteins pits and irf2bpl respectively, a human *in vitro* model was developed, elucidating the role of *de novo* nonsense mutations Glu172*, Tyr173*, Arg188*, and the missense variant A708Fs59 in the haploinsufficiency of the IRF2BPL gene. Healthy controls and patient fibroblasts were directly converted into induced neurons (iNs) confirming the cellular localization of IRF2BPL to the nucleus with a vague signal in the cytoplasm of the patient iNs. The protein expression level was remarkably similar between healthy controls and patients with NEDAMSS with an exception for the adult patient cell line, containing the Arg188* variant, which displayed a significant reduction in the expression level of full-length IRF2BPL. Yet, a general decrease in neurite length was noticed in patient iNs (Sinha Ray et al., 2022), suggesting an inefficient migration and maturation into axons and dendrites, affecting the connection and communication with other neurons (Prem et al., 2020), leading to neuronal loss (Marcogliese et al., 2022).

Since the CNS is a highly complex network with a great variety of cell populations of both neuronal and non-neuronal cells, hereof glial cells with the most predominant subtype being astrocytes. The cellular phenotype of astrocytes in NEDAMSS was therefore studied by the induction of healthy controls and patient fibroblasts into astrocytes (iAs). As a result, patient iAs exhibited a distinct phenotype from the healthy iAs with altered morphology, abnormal mitochondrial activity, and greater GFAP expression, which is frequently linked to activated astrocytes. Besides that, the IRF2BPL protein was primarily mislocalized as aggregates in the cytoplasm in the form of dimers between the truncated and full-length protein isoforms in the four patient iAs compared to the healthy control, whereof the expression of IRF2BPL was predominantly located to the nucleus. This was additionally confirmed by a similar transcription level of truncated IRF2BPL mRNA to endogenous full-length IRF2BPL mRNA levels in the patient iAs, displaying stable expression for translation. Consistent with a single exon transcript there is no protein decay of the nonsense variants upon the introduction of a premature stop codon and transcription aberration (Marcogliese et al., 2018; Qian et al., 2021), resulting in the dissociation between the truncated and the endogenous full-length IRF2BPL protein and its mislocalization in the cytoplasm (Sinha Ray et al., 2022). Co-culturing of mouse neurons and patient iAs, exhibiting an abnormal morphology and alteration in the GFAP expression level were inadequate to support the survival of mouse neurons compared to healthy iAs, indicating insufficient maturation and plasticity of synaptic transmission (Perea et al., 2009; Prem et al., 2020; Liu et al., 2021).

## 5 Treatments and therapeutic strategies for NEDAMSS

Nevertheless, the treatment of IRF2BPL-related disorders based on the current understanding of the biological function of IRF2BPl has been limited, as the main focus is still on alleviating the patient’s symptoms by symptomatic multidisciplinary management rather than curing them. Controlling of the seizures has thereby been accomplished by the use of antiseizure medication, including sodium valproate, perampanel levetiracetam, sulthiame, topiramate, lamotrigine, zonisamide, and carbamazepine (Pisano et al., 2022; Gardella et al., 2023; Khan et al., 2023), whereas patients with epileptic manifestation received levetiracetam and clonazepam, lamotrigine, carbamazepine, oxcarbazepine, topiramate, valproic acid, zonisamide, and perampanel (Pisano et al., 2022; Khan et al., 2023). Further, levetiracetam, topiramate, lamotrigine, clobazam, and high doses of primidone partially improved the myoclonus of patients (Costa et al., 2023; Gardella et al., 2023).

Recently, a new potential candidate for the treatment of NEDAMSS was discovered. CuII(atsm) (diacetylbis(4-methylthiosemicarbazonato) copperII) (CuATSM) is a small molecular weight, artificial, orally bioavailable drug that can pass the human blood–brain barrier. CuATSM is thought to exert neuroprotective effects in disease-affected regions of the CNS in ALS, PD, and more (Soon et al., 2011; Hung et al., 2012; Kuo et al., 2019;Nikseresht et al., 2020; Sinha Ray et al., 2022). The literature suggests that CuATSM’s potential mechanisms involve the restoration of mitochondrial function, including the correction of increased fractionation and mislocalization of mitochondria observed in NEDAMSS patient cells (Sinha Ray et al., 2022). The drug is safe for use in humans and is currently in clinical trials for hypoxic imaging, ALS treatment (Dennys et al., 2023), and Parkinson’s disease (Nikseresht et al., 2020; Sinha Ray et al., 2022). In the phase I clinical trial of ALS, the drug slowed down disease progression and improved the respiratory and cognitive function of the patients (Sinha Ray et al., 2022). However, its exact mechanism of action is still unknown (Sinha Ray et al., 2022; Dennys et al., 2023). Sinha Ray et al. (2022) showed that treatment of patient-derived induced iAs with 1 μM CuATSM for four consecutive days, before neuron-glia co-culture, significantly increased neuronal survival. However, it did not rescue the mislocalization of IRF2BPL to the cytoplasm in patient iAs. Further, CuATSM treatment reduced the elevated levels of mitochondrial respiration to normal control levels in three out of four patient iAs. In addition, the transcriptional profile of treated iAs shows upregulation of metal ion homeostasis pathways which is important for cellular viability. The authors suggest that selective metal homeostasis could improve mitochondrial respiration resulting in neuroprotection. In their Drosophila model of heterozygous PitsTG4/+ flies, they observed that the ones treated with CuATSM displayed decreased time to climb, indicating that the drug is at least partially neuroprotective in ameliorating climbing defects at 35 days after eclosion (Sinha Ray et al., 2022).

Within the last decade, gene therapies gained attention as a promising tool to not only treat but also cure the patient’s symptoms. Gene therapies provide the opportunities to introduce, remove, or change DNA and/or RNA in patient cells to treat the genetic disorder (Nerkar and Chakraborthy, 2021). Several types of gene therapies are currently clinically available, including Short synthetic antisense oligonucleotides (ASOs) approved to treat spinal muscular atrophy (Nusinersen, Spinraza^™^) by modulating the splicing of the SMN2 gene to increase the quantity of the stable full-length SMN protein (Jensen et al., 2021). Another example is Tofersen (Qalsody^™^) for the treatment of amyotrophic lateral sclerosis (ALS) by inhibiting transcription of the mutated superoxide dismutase 1 (SOD1) gene (Blair, 2023). Gene Replacement Therapies involve the introduction of a healthy gene copy to complement a defective one such as Zolgensma^®^ which is FDA-approved for spinal muscular atrophy (Jensen et al., 2021). Recently, the first gene editing therapy CASGEVY^®^ has been approved for the treatment of sickle cell disease, through CRISPR-Cas9 targeting and correction of BCL11A in patient hematopoietic stem, followed by the infusion back into the patient (Philippidis, 2024). Regarding gene editing strategies, nuclease-independent ones such as Base- and Prime Editing have advantages over double-strand break (DSB)-based strategies by minimizing the probability of unwanted large-scale alterations of an organism’s genome with the possibility of improving gene editing efficiencies (Alves et al., 2023).

Base editors (BEs) are in principle an excellent tool for the treatment of genetic diseases like NEDAMSS and have already been reviewed extensively (Anzalone et al., 2020; Porto et al., 2020; Newby and Liu, 2021; Tran et al., 2022; Yu et al., 2022). BEs have several advantages over conventional CRISPR-Cas9-associated methods. Since they are associated with base excision repair (BER) and mismatch repair (MMR) that occur extensively in most cell-cycle phases, BEs can be applied in non-dividing cell populations like neurons and astrocytes that are affected in patients suffering from NEDAMSS. Further, they do not require a donor template, making them less toxic and easier to deliver. So far, there are four ongoing clinical trials with BEs, but none of them are targeting the CNS (Porto and Komor, 2023).

Adenine base editors (ABEs) (Gaudelli et al., 2017), which convert Adenine (A) to Guanine (G), are of particular interest in the field of precision medicine. This is because mutations of a G•C base pair to an A•T base pair, which can be corrected by adenine base editing, represent ~47% of disease-associated point mutations. Regarding NEDAMSS, ~42% of mutations, representing 16 out of 34 reported patients, could theoretically be corrected using ABEs. Recently Cytosine (C) to Guanine Base Editors (CGBEs) (Chen et al., 2021) have been developed, which catalyze the conversion of a C•G to a G•C base pair. The application of CGBEs, can therefore correct three mutations affecting five NEDAMSS patients. However, there are only a few studies investigating CGBEs and they are suspected to generate double-strand breaks causing deletion, transversion, and translocation, resulting in their less favorable utilization in gene therapy (Huang et al., 2024).

Prime editors (PEs) on the other hand consist of a Cas9 nickase fused to a reverse transcriptase enzyme, along with a prime editing guide RNA (pegRNA), providing them with the ability to insert or delete SNPs or smaller indels. It has been studied in different cell types and diseases which are reviewed here (Newby and Liu, 2021; Chen and Liu, 2023; Zhao et al., 2023). Theoretically, PEs can correct all possible base-to-base conversions and indel which is 89% of all human pathogenic genetic variants (Anzalone et al., 2019; Chen and Liu, 2023). However, PE is a relatively new technology and the editing efficiency is usually low. Further, there are safety concerns regarding the off-target activity of the reverse transcriptase.

## 6 Discussion

One of the underlying reasons for the absence of therapeutic avenues for NEDAMSS is the new and ultra-rare nature of the disease. Hence, understanding the specific molecular characteristics of the disorder during neurodevelopment would be of invaluable significance for gaining knowledge of the pathogenic process related to the neurodegenerative aspect of the disease and in particular to the treatment of NEDAMSS. Yet, neurological disorders remain a challenging task due to the lack of patient-derived brain tissues to investigate and recapitulate the phenotypes associated with the function of IRF2BPL during early human neurodevelopment, as patient biopsies usually represent the endpoint of the disease and do not reveal the causative mechanisms (Sidhaye and Knoblich, 2021). Common questions remain unsolved regarding the examination of early neurodevelopmental components such as the initial mislocalization and aggregation of mutant IRF2BPL protein, plausible defects in neuro progenitor cell polarity and differentiation, and changes in mitosis or cell cycle progression. Therefore, the majority of model organisms predominantly rely on evolutionary distanced animals, such as Drosophila, zebrafish, and rodents to mimic complex biological processes, as they are significantly simpler, and provide greater accessibility for gene manipulation and insight into signaling pathways in the initial stages of development (Jennings, 2011; Adhish and Manjubala, 2023). To date, rodent and mouse models in particular are considered the golden standard for research, due to the sharing of the common brain functions, making them invaluable models for behavioral studies from the onset to the end stage of the disorder (Gurumurthy and Lloyd, 2019). Unfortunately, no rodent model system has been generated to study the behavioral pattern of the various pathogenic variants in IRF2BPL, limiting the correlation of specific mutations and their location to visible phenotypic traits.

Nevertheless, continued advancement in stem cell technology has paved the way for three-dimensional (3D) brain organoids, which can recapitulate the spatiotemporal organization and function of various cell populations found in the human brain during neurodevelopment (Jacob et al., 2021; Shafique, 2022). Especially, patient-derived brain organoids can in the future provide insights into the individual pathogenic variants of IRF2BPL’s effect on the downstream transcriptomic mechanisms, aggregation, and/or mislocalization, resulting in crucial information on pathways affected by the disease that can be pharmacologically targeted, which has not possible with animal models. To achieve this goal, a tight integration of clinical and basic research is essential.

While new drugs like CuATSM might slow down the disease progression and improve patient behavior, only gene editing has the potential to permanently correct the disease-causing mutations. However, due to the broad heterogeneity of mutations in IRF2BPL, editing strategies must be developed specifically for each patient. Even though precision medicine is a rising field, it means a lot of effort and high costs.

In addition to the common challenges regarding gene therapy (Porto et al., 2020; Newby and Liu, 2021; Porto and Komor, 2023), delivery methods to the human brain that can pass the human blood–brain barrier remain a challenge. Also, it is not clear yet, which is the latest time point of intervention, meaning if correction of the mutations in the developed brain restores the healthy phenotype.

Taken together, a lot of research has been done since the diagnosis of the first NEDAMSS patient in 2018. The growing awareness of the disease and improvements in the diagnosis constantly lead to the identification of new patients with new phenotypes (Vanagunas and Pena, 2024). Further, the development of animal models as well as 3D brain organoids will help fill the gaps of IRF2BPL in neurodevelopment and accelerate the generation of new drugs and gene therapies.

## Author contributions

DB: Writing – original draft, Writing – review & editing. LB: Writing – original draft, Writing – review & editing. SR: Writing – original draft, Writing – review & editing. RK: Writing – original draft, Writing – review & editing. PL: Writing – original draft, Writing – review & editing.

## References

[ref1] Adhish M. Manjubala I. (2023). Effectiveness of zebrafish models in understanding human diseases—a review of models. Heliyon 9:e14557. doi: 10.1016/j.heliyon.2023.e14557, PMID: 36950605 PMC10025926

[ref2] Alves C. R. R. Ha L. L. Yaworski R. Lazzarotto C. R. Christie K. A. Reilly A.. (2023). Base editing as a genetic treatment for spinal muscular atrophy. bioRxiv. doi: 10.1101/2023.01.20.524978, PMID: 36711797 PMC9882371

[ref3] Anzalone A. V. Koblan L. W. Liu D. R. (2020). Genome editing with CRISPR–Cas nucleases, base editors, transposases and prime editors. Nat. Biotechnol. 38, 824–844. doi: 10.1038/s41587-020-0561-9 32572269

[ref4] Anzalone A. V. Randolph P. B. Davis J. R. Sousa A. A. Koblan L. W. Levy J. M.. (2019). Search-and-replace genome editing without double-strand breaks or donor DNA. Nature 576, 149–157. doi: 10.1038/s41586-019-1711-4, PMID: 31634902 PMC6907074

[ref5] Blair H. A. (2023). Tofersen: first approval. Drugs 83, 1039–1043. doi: 10.1007/s40265-023-01904-6, PMID: 37316681

[ref6] Chen P. J. Liu D. R. (2023). Prime editing for precise and highly versatile genome manipulation. Nat. Rev. Genet. 24, 161–177. doi: 10.1038/s41576-022-00541-1, PMID: 36344749 PMC10989687

[ref7] Chen L. Park J. E. Paa P. Rajakumar P. D. Prekop H. T. Chew Y. T.. (2021). Programmable C:G to G:C genome editing with CRISPR-Cas9-directed base excision repair proteins. Nat. Commun. 12:1384. doi: 10.1038/s41467-021-21559-9, PMID: 33654077 PMC7925527

[ref8] Costa C. Oliver K. L. Calvello C. Cameron J. M. Imperatore V. Tonelli L.. (2023). IRF2BPL: a new genotype for progressive myoclonus epilepsies. Epilepsia 64, e164–e169. doi: 10.1111/epi.17557, PMID: 36810721

[ref9] Dennys C. N. Roussel F. Rodrigo R. Zhang X. Sierra Delgado A. Hartlaub A.. (2023). CuATSM effectively ameliorates ALS patient astrocyte-mediated motor neuron toxicity in human in vitro models of amyotrophic lateral sclerosis. Glia 71, 350–365. doi: 10.1002/glia.24278, PMID: 36213964 PMC10092379

[ref10] ENSEMBL IRF2BPL. Available at: https://www.ensembl.org/Homo_sapiens/Gene/Summary?db=core;g=ENSG00000119669;r=14:77024543-77028708;t=ENST00000238647 (Accessed: 29 April 2024)

[ref11] Gardella E. Michelucci R. Christensen H. M. Fenger C. D. Reale C. Riguzzi P.. (2023). IRF2BPL as a novel causative gene for progressive myoclonus epilepsy. Epilepsia 64:17634. doi: 10.1111/epi.17634 37114479

[ref12] Gaudelli N. M. Komor A. C. Rees H. A. Packer M. S. Badran A. H. Bryson D. I.. (2017). Programmable base editing of T to G C in genomic DNA without DNA cleavage. Nature 551, 464–471. doi: 10.1038/nature24644, PMID: 29160308 PMC5726555

[ref13] Ginevrino M. Battini R. Nuovo S. Simonati A. Micalizzi A. Contaldo I.. (2020). A novel IRF2BPL truncating variant is associated with endolysosomal storage. Mol. Biol. Rep. 47, 711–714. doi: 10.1007/s11033-019-05109-7, PMID: 31583567

[ref14] Gurumurthy C. B. Lloyd K. C. K. (2019). Generating mouse models for biomedical research: technological advances. Dis. Model. Mech. 12:29462. doi: 10.1242/dmm.029462, PMID: 30626588 PMC6361157

[ref15] Higashimori A. Dong Y. Zhang Y. Kang W. Nakatsu G. Ng S. S. M.. (2018). Forkhead box F2 suppresses gastric cancer through a novel FOXF2-IRF2BPL-β-catenin signaling axis. Cancer Res. 78, 1643–1656. doi: 10.1158/0008-5472.CAN-17-2403, PMID: 29374064

[ref16] Huang M. E. Qin Y. Shang Y. Hao Q. Zhan C. Lian C.. (2024). C-to-G editing generates double-strand breaks causing deletion, transversion and translocation. Nat. Cell Biol. 26, 294–304. doi: 10.1038/s41556-023-01342-2, PMID: 38263276

[ref17] Hung L. W. Villemagne V. L. Cheng L. Sherratt N. A. Ayton S. White A. R.. (2012). The hypoxia imaging agent CuII(atsm) is neuroprotective and improves motor and cognitive functions in multiple animal models of Parkinson’s disease. J. Exp. Med. 209, 837–854. doi: 10.1084/jem.20112285, PMID: 22473957 PMC3328361

[ref18] Jacob F. Schnoll J. G. Song H. Ming G. (2021). Building the brain from scratch: engineering region-specific brain organoids from human stem cells to study neural development and disease 142, 477–530. doi: 10.1016/bs.ctdb.2020.12.011, PMID: 33706925 PMC8363060

[ref19] Jennings B. H. (2011). Drosophila—a versatile model in biology & medicine. Mater. Today 14, 190–195. doi: 10.1016/S1369-7021(11)70113-4

[ref20] Jensen T. L. Gøtzsche C. R. Woldbye D. P. D. (2021). Current and future prospects for gene therapy for rare genetic diseases affecting the brain and spinal cord. Front. Mol. Neurosci. 14:695937. doi: 10.3389/fnmol.2021.695937, PMID: 34690692 PMC8527017

[ref21] Jiang S. Zhang M. Sun J. Yang X. (2018). Casein kinase 1α: biological mechanisms and theranostic potential. Cell Commun. Signal. 16:23. doi: 10.1186/s12964-018-0236-z, PMID: 29793495 PMC5968562

[ref22] Khan W. J. Maqsood H. Younus S. (2023). Novel IRF2BPL gene mutation manifesting as a broad spectrum of neurological disorders: a case report. BMJ Neurolo. Open 5:e000459. doi: 10.1136/bmjno-2023-000459, PMID: 37649702 PMC10462932

[ref23] Kuo M. T. H. Beckman J. S. Shaw C. A. (2019). Neuroprotective effect of CuATSM on neurotoxin-induced motor neuron loss in an ALS mouse model. Neurobiol. Dis. 130:104495. doi: 10.1016/j.nbd.2019.104495, PMID: 31181282

[ref24] Liu X. Ying J. Wang X. Zheng Q. Zhao T. Yoon S.. (2021). Astrocytes in neural circuits: key factors in synaptic regulation and potential targets for neurodevelopmental disorders. Front. Mol. Neurosci. 14:729273. doi: 10.3389/fnmol.2021.729273, PMID: 34658786 PMC8515196

[ref25] Marcogliese P.C. Dutta D. Ray S.S. Dang N.D.P. Zuo Z. Wang Y., (2022). Loss of IRF2BPL impairs neuronal maintenance through excess Wnt signaling. Available at: https://www.science.org 10.1126/sciadv.abl5613 PMC8769555 35044823

[ref26] Marcogliese P. C. Shashi V. Spillmann R. C. Stong N. Rosenfeld J. A. Koenig M. K.. (2018). IRF2BPL is associated with neurological phenotypes. Am. J. Hum. Genet. 103, 245–260. doi: 10.1016/j.ajhg.2018.07.006, PMID: 30057031 PMC6081494

[ref27] Nerkar A. G. Chakraborthy G. S. (2021). Gene therapy. Curr. Trends Pharmacy Pharmac. Chem. 3, 15–18. doi: 10.18231/j.ctppc.2021.005

[ref28] Newby G. A. Liu D. R. (2021). In vivo somatic cell base editing and prime editing. Mol. Ther. 29, 3107–3124. doi: 10.1016/j.ymthe.2021.09.002, PMID: 34509669 PMC8571176

[ref29] Nikseresht S. Hilton J. B. W. Kysenius K. Liddell J. R. Crouch P. J. (2020). Copper-ATSM as a treatment for ALS: support from mutant SOD1 models and beyond. Life 10:271. doi: 10.3390/life10110271, PMID: 33158182 PMC7694234

[ref30] Perea G. Navarrete M. Araque A. (2009). Tripartite synapses: astrocytes process and control synaptic information. Trends Neurosci. 32, 421–431. doi: 10.1016/j.tins.2009.05.001, PMID: 19615761

[ref31] Philippidis A. (2024). CASGEVY makes history as FDA approves first CRISPR/Cas9 genome edited therapy. Hum. Gene Ther. 35, 1–4. doi: 10.1089/hum.2023.29263.bfs, PMID: 38231658

[ref32] Pisano S. Melis M. Figorilli M. Polizzi L. Rocchi L. Giglio S.. (2022). Neurological phenomenology of the IRF2BPL mutation syndrome: analysis of a new case and systematic review of the literature. Seizure 99, 12–15. doi: 10.1016/j.seizure.2022.04.010, PMID: 35525099

[ref33] Porto E. M. Komor A. C. (2023). In the business of base editors: evolution from bench to bedside. PLoS Biol. 21:e3002071. doi: 10.1371/journal.pbio.3002071, PMID: 37043430 PMC10096463

[ref34] Porto E. M. Komor A. C. Slaymaker I. M. Yeo G. W. (2020). Base editing: advances and therapeutic opportunities. Nat. Rev. Drug Discov. 19, 839–859. doi: 10.1038/s41573-020-0084-6, PMID: 33077937 PMC7721651

[ref35] Prem S. Millonig J. H. DiCicco-Bloom E. (2020). Dysregulation of neurite outgrowth and cell migration in autism and other neurodevelopmental disorders, 109–153. 10.1007/978-3-030-45493-7_5 32578146

[ref36] Prilop L. Buchert R. Woerz S. Gerloff C. Haack T. B. Zittel S. (2020). IRF2BPL mutation causes nigrostriatal degeneration presenting with dystonia, spasticity and keratoconus. Parkinsonism Relat. Disorders 79, 141–143. doi: 10.1016/j.parkreldis.2020.03.030, PMID: 32291157

[ref37] Qian X. Liu X. Y. Zhu Z. Y. Wang S. G. Song X. X. Chen G.. (2021). Neurodevelopmental disorder caused by a truncating de novo variant of IRF2BPL. Seizure 84, 47–52. doi: 10.1016/j.seizure.2020.11.006, PMID: 33278788

[ref38] Rampazzo A. Pivotto F. Occhi G. Tiso N. Bortoluzzi S. Rowen L.. (2000). Characterization of C14orf4, a novel intronless human gene containing a polyglutamine repeat, mapped to the ARVD1 critical region. Biochem. Biophys. Res. Commun. 278, 766–774. doi: 10.1006/bbrc.2000.3883, PMID: 11095982

[ref39] Sabitha K. R. Shetty A. K. Upadhya D. (2021). Patient-derived iPSC modeling of rare neurodevelopmental disorders: molecular pathophysiology and prospective therapies. Neurosci. Biobehav. Rev. 121, 201–219. doi: 10.1016/j.neubiorev.2020.12.025, PMID: 33370574 PMC7962756

[ref40] Shafique S. (2022). Stem cell-based region-specific brain organoids: novel models to understand neurodevelopmental defects. Birth Defects Res. 114, 1003–1013. doi: 10.1002/bdr2.2004, PMID: 35332709

[ref41] Shelkowitz E. Singh J. K. Larson A. Elias E. R. (2019). IRF2BPL gene mutation: expanding on neurologic phenotypes. Am. J. Med. Genet. A 179, 2263–2271. doi: 10.1002/ajmg.a.61328 31432588

[ref42] Shimono Y. Murakami H. Hasegawa Y. Takahashi M. (2000). RET finger protein is a transcriptional repressor and interacts with enhancer of Polycomb that has dual transcriptional functions. J. Biol. Chem. 275, 39411–39419. doi: 10.1074/jbc.M006585200, PMID: 10976108

[ref43] Sidhaye J. Knoblich J. A. (2021). Brain organoids: an ensemble of bioassays to investigate human neurodevelopment and disease. Cell Death Diff. 28, 52–67. doi: 10.1038/s41418-020-0566-4, PMID: 32483384 PMC7853143

[ref44] Sinha Ray S. Dutta D. Dennys C. Powers S. Roussel F. Lisowski P.. (2022). Mechanisms of IRF2BPL-related disorders and identification of a potential therapeutic strategy. Cell Rep. 41:111751. doi: 10.1016/j.celrep.2022.111751, PMID: 36476864

[ref45] Skorvanek M. Dusek P. Rydzanicz M. Walczak A. Kosinska J. Kostrzewa G.. (2019). Neurodevelopmental disorder associated with IRF2BPL gene mutation: expanding the phenotype? Parkinsonism Relat. Disord. 62, 239–241. doi: 10.1016/j.parkreldis.2019.01.017, PMID: 30733140

[ref46] Soon C. P. W. Donnelly P. S. Turner B. J. Hung L. W. Crouch P. J. Sherratt N. A.. (2011). Diacetylbis(N(4)-methylthiosemicarbazonato) copper(II) (CuII(atsm)) protects against Peroxynitrite-induced Nitrosative damage and prolongs survival in amyotrophic lateral sclerosis mouse model. J. Biol. Chem. 286, 44035–44044. doi: 10.1074/jbc.M111.274407, PMID: 22033929 PMC3243559

[ref47] The Human Protein Atlas. Available at: https://www.proteinatlas.org/ENSG00000119669-IRF2BPL/tissue (Accessed: 29 April 2024)

[ref48] Tran M. T. N. Kc R. Hewitt A. W. (2022). A taxonomic and phylogenetic classification of diverse base editors. CRISPR J. 5, 311–328. doi: 10.1089/crispr.2021.0095, PMID: 35244489

[ref49] Tran Mau-Them F. Guibaud L. Duplomb L. Keren B. Lindstrom K. Marey I.. (2019). De novo truncating variants in the intronless IRF2BPL are responsible for developmental epileptic encephalopathy. Genet. Med. 21, 1008–1014. doi: 10.1038/s41436-018-0143-0, PMID: 30166628

[ref50] Vanagunas T. Pena L. (2024). P132: expansion of the IRF2BPL-related disorder phenotype: initial updates from natural history study. Genet. Med. Open 2:101029. doi: 10.1016/j.gimo.2024.101029

[ref51] Yu S. Y. Birkenshaw A. Thomson T. Carlaw T. Zhang L. H. Ross C. J. D. (2022). Increasing the targeting scope of CRISPR Base editing system beyond NGG. CRISPR J. 5, 187–202. doi: 10.1089/crispr.2021.0109, PMID: 35238621

[ref52] Zhao Z. Shang P. Mohanraju P. Geijsen N. (2023). Prime editing: advances and therapeutic applications. Trends Biotechnol. 41, 1000–1012. doi: 10.1016/j.tibtech.2023.03.004 37002157

